# Development and validation of the competing risk nomogram and risk classification system for predicting cancer-specific mortality in patients with cervical adenosquamous carcinoma treated via radical hysterectomy

**DOI:** 10.17305/bb.2024.11217

**Published:** 2024-11-06

**Authors:** Jianying Yi, Jie Chen, Xi Cao, Lili Pi, Chunlei Zhou, Zhili Liu, Hong Mu

**Affiliations:** 1 Department of Clinical Laboratory, Tianjin First Central Hospital, School of Medicine, Nankai University, Tianjin, China; 2 Department of Clinical Laboratory, The Third Central Hospital, Tianjin, China; 3 Tianjin Key Laboratory of Extracorporeal Life Support for Critical Diseases, Tianjin, China; 4 Artificial Cell Engineering Technology Research Center, Tianjin, China; 5 Tianjin Institute of Hepatobiliary Disease, Tianjin, China

**Keywords:** Competing risk nomogram, cervical adenosquamous carcinoma, cancer-specific mortality, radical hysterectomy, SEER

## Abstract

In this study, we established and validated a competing risk nomogram for predicting the cumulative incidence of cervical adenosquamous carcinoma (ASC)-specific death in patients undergoing radical hysterectomy. Patients diagnosed with ASC between 2010 and 2019 were retrieved from the Surveillance, Epidemiology, and End Results database. The cumulative incidence function for various variables influencing ASC-specific mortality was computed. A Fine–Gray competing risk model was used to identify independent predictors, formulating a competing risk nomogram. A multivariate Cox proportional hazards model was also applied for comparative analysis. The performance of the nomogram was assessed using metrics, such as the concordance index, receiver operating characteristic curves, calibration curves, and decision curve analysis. A corresponding risk classification system was constructed based on nomogram-derived scores. Factors, such as advanced age, racial background (Black race), higher tumor grade, increased tumor size, advanced TNM stage, and receipt of radiotherapy without chemotherapy, were found to be positively associated with elevated ASC-specific mortality. Additionally, age, T stage, M stage, and chemotherapy were identified as independent predictors correlated with ASC-specific mortality. The established nomogram exhibited accurate discriminatory capabilities and superior net benefits compared to the traditional TNM staging system. Additionally, the high-risk group consistently demonstrated higher probabilities of ASC-specific death in both the training and validation sets. The developed nomogram proficiently quantified the incidence of ASC-specific death in patients subjected to radical hysterectomy for ASC. This tool could help clinicians in formulating personalized treatment strategies and devising follow-up protocols.

## Introduction

### Background

Despite concentrated efforts spanning recent decades to diminish the incidence of cervical cancer through screening initiatives and the implementation of human papillomavirus (HPV) vaccination, cervical cancer persists as the fourth most prevalent cancer among women globally, contributing significantly to cancer-related mortality [1–3]. Cervical adenosquamous carcinoma (ASC), a rare histological subtype of cervical cancer that accounts for only 3%–10% of all cervical malignancies, has shown an increasing trend over the years, particularly among postmenopausal women [4, 5]. Characterized by a composite nature involving squamous cell carcinoma (SCC) and adenocarcinoma, ASC presents a less favorable prognosis compared to other cervical cancer types, which is attributed to a more aggressive lymph node invasion and a greater propensity for distant metastases [6, 7]. Yokoi et al. [8] observed significantly shorter progression-free survival (PFS) (*P* ═ 0.002) and overall survival (OS) (*P* ═ 0.004) in ASC patients compared to those with cervical SCC, establishing histological ASC as an independent prognostic factor for PFS through multivariate analysis. Today the inherent resistance of the cervix to therapeutic drugs, coupled with the limited success of radiotherapy, underscores the continued prominence of radical hysterectomy as the primary treatment modality for early to mid-stage ASC. Retrospective studies have consistently affirmed the pivotal role of radical hysterectomy in extending the survival of cervical cancer patients [9, 10]. Liu et al. [11] reported a significant trend favoring radical hysterectomy over radio-chemotherapy for IB1-IIA2 cervical cancer, evidencing improved OS (84.6% vs 76.1%, *P* < 0.001) and disease-free survival (DFS) (81.5% vs 75.1%, *P* < 0.001). Nevertheless, scant attention has been directed toward investigating the prognosis of ASC patients undergoing radical hysterectomy. Reports examining the survival of ASC often rely on Kaplan–Meier analyses and Cox regression models [12–14]. However, both models encompass two outcome events, namely, death or censoring, without accounting for competing risk events. The presence of non-specific mortality stemming from these competing risks may lead to an overestimation of the probability of ASC-specific death. Moreover, prognostic nomograms based on competing risk analysis can incorporate numerous clinical and demographic factors into a visual evaluation model for predicting the prognosis of specific events [15, 16]. Nevertheless, to date, no studies have formulated a competing risk model to predict factors influencing cancer-specific mortality in ASC patients subjected to radical hysterectomy.

### Objectives

The infrequent occurrence of ASC and the restricted number of cases in most studies make it challenging to accurately portray the survival of ASC patients. To provide a quantitative tool for predicting ASC-specific mortality in patients undergoing radical hysterectomy for ASC, thereby enhancing clinical decision making, we developed and validated a novel competing risk nomogram using a comprehensive dataset from the Surveillance, Epidemiology, and End Results (SEER) database.

## Materials and methods

### Study cohorts and selection criteria

**Figure 1.**
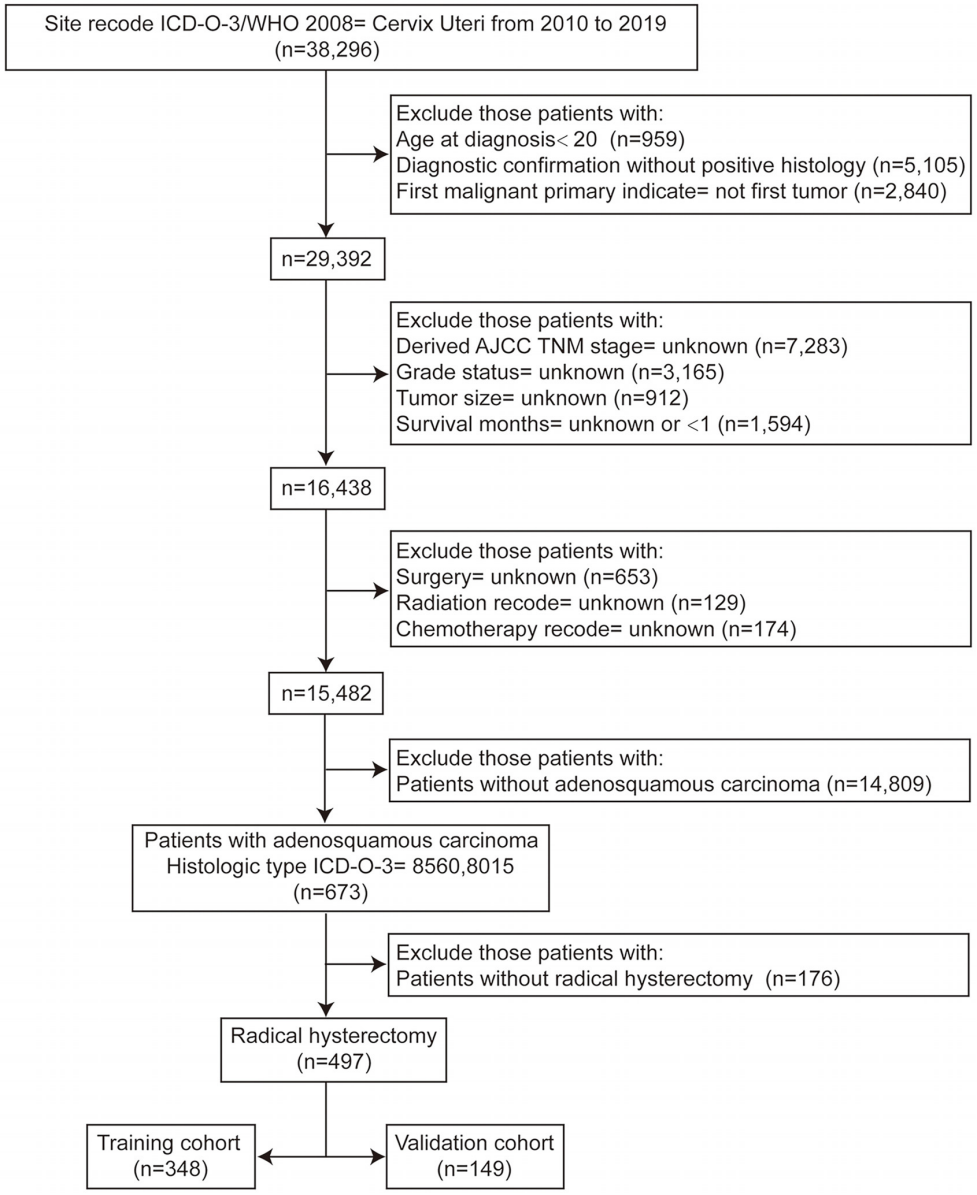
Flowchart illustrating the process of patient selection.

In this retrospective study, we extracted data from the public SEER database, accession number 11761-Nov2021, encompassing patients pathologically diagnosed with ASC between 2010 and 2019. The inclusion criteria were as follows: (1) a pathological diagnosis of ASC (site recode ICD-O-3/WHO2008 ═ Cervix Uteri and histologic type ICD-O-3 ═ 8560, 8015) from 2010 to 2019; (2) ASC as the sole primary malignancy or the initial primary malignancy; (3) age ≥ 20 years at the time of diagnosis; (4) availability of demographic data and tumor characteristics. The exclusion criteria were: (1) individuals diagnosed post-mortem or by death certificate evaluation; (2) cases with incomplete TNM stage records, missing tumor grade status, or unknown tumor size records; (3) survival < 1 month or unknown survival data; and (4) cases lacking information about treatment. A total of 497 ASC patients who underwent radical hysterectomy met the inclusion criteria and constituted the study cohort. A comprehensive flowchart outlining the patient selection process is depicted in Figure 1.

### Data collection

To ensure accuracy and comprehensiveness, we included all variables from the SEER database in our analysis, encompassing demographic characteristics, tumor features, treatment details, and patient survival data. These variables covered factors, such as age at diagnosis, race, histological grade, tumor size, TNM stage, surgery, radiotherapy, chemotherapy, marital status, vital status, survival time, and cause of death. After screening, no individuals reported their marital status as “Unknown” or “Blank.” We consolidated “Married” (including common law) into a single married category, while all other statuses—i.e., Single (never married), Separated, Divorced, Widowed, and Unmarried or domestic partner (same-sex or opposite-sex, or unregistered)—were grouped into a single category.

Subsequently, these variables were incorporated into both univariable and multivariable competing risk models to identify independent predictors associated with ASC-specific mortality. The final follow-up status included ASC-specific mortality, mortality from other causes, and censoring. ASC-specific mortality was defined as death resulting directly from primary ASC. Mortality from other causes denoted death due to any factors unrelated to ASC. Individuals who remained alive at the conclusion of the last follow-up were categorized as censored events.

### Ethical statement

Given the publicly available and de-identified nature of the SEER datasets, our study was exempt from medical ethics review and did not require informed consent from individuals.

### Statistical analysis

The chi-square test or Fisher’s exact test was performed to examine group differences. Recognizing that mortality from causes unrelated to ASC could serve as competing events for ASC-specific mortality, we computed the cumulative incidence function (CIF) for various variables on ASC-specific mortality. Gray’s test was then used to assess significant disparities in CIF values across subgroups. Significant variables were identified in the univariable competing risk analysis using Gray’s proportional subdistribution hazard model. Subsequently, these variables were incorporated into a multivariable Fine and Gray analysis to determine independent predictors associated with ASC-specific mortality. A reverse stepwise stop rule was applied to further screen the variables. The model quality was evaluated using the Akaike information criterion (AIC) as the final selection criterion, with the model displaying the lowest AIC value identified as the optimal model. A multivariate Cox proportional hazards model was also applied for comparative analysis.

Finally, a competing risk nomogram was developed using the predictors derived from the final multivariable competing risk model. Two critical aspects of the nomogram’s performance—accuracy and discrimination power—were evaluated using the concordance index (C-index), receiver operating characteristic (ROC) curves, and calibration curves. To mitigate potential overfitting, calibration was estimated by comparing predicted probabilities with actual outcomes using 1000 bootstrap samples. Additionally, decision curve analysis (DCA) was applied to assess the clinical utility of the competing risk nomogram by quantifying net benefits across various threshold probabilities. Based on dichotomous values of nomogram-derived scores, all ASC patients treated with radical hysterectomy were categorized into high-risk (total score ≥ 206) and low-risk (total score < 206) groups. The CIF curves of these groups were then plotted to validate the prognostic value of the nomogram.

All statistical analyses and visualizations were conducted using SPSS 25.0 and R software (version 4.2.2), utilizing packages, such as table1, survival, survminer, rms, mstate, cmprsk, regplot, nomogramFormula, and survivalROC. A *P* value < 0.05 was deemed statistically significant.

**Table 1:** Baseline clinicopathological features of ASC patients treated with radical hysterectomy in the training and validation cohort

**Variables**	**Training cohort**	**Validation cohort**	***P^†^* value**
	**(*N* ═ 348)**	**(*N* ═ 149)**	
*Age (year)*			0.968
≤ 45	148 (42.5%)	62 (41.6%)	
46–59	110 (31.6%)	49 (32.9%)	
≥ 60	90 (25.9%)	38 (25.5%)	
*Race*			0.056
Black	28 (8.0%)	20 (13.4%)	
White	272 (78.2%)	102 (68.5%)	
Other	48 (13.8%)	27 (18.1%)	
*Grade*			0.986
I	169 (48.6%)	73 (49.0%)	
II	56 (16.1%)	24 (16.1%)	
III	79 (22.7%)	35 (23.5%)	
IV	44 (12.6%)	17 (11.4%)	
*Tumor size (cm)*			0.996
≤ 2	90 (25.9%)	38 (25.5%)	
2.1–3.9	116 (33.3%)	50 (33.6%)	
≥ 4	142 (40.8%)	61 (40.9%)	
*T stage*			0.311
T1 + T2	289 (83.0%)	118 (79.2%)	
T3 + T4	59 (17.0%)	31 (20.8%)	
*N stage*			0.201
N0	305 (87.6%)	124 (83.2%)	
N1	43 (12.4%)	25 (16.8%)	
*M stage*			0.222
M0	330 (94.8%)	137 (91.9%)	
M1	18 (5.2%)	12 (8.1%)	
*Radiotherapy*			0.358
None	119 (34.2%)	58 (38.9%)	
Yes	229 (65.8%)	91 (61.1%)	
*Chemotherapy*			0.998
None	162 (46.6%)	70 (47.0%)	
Yes	186 (53.4%)	79 (53.0%)	
*Marital status*			0.493
Single	190 (54.6%)	76 (51.0%)	
Married	158 (45.4%)	73 (49.0%)	

*P*^†^ indicates chi-square test or Fisher’s exact test. ASC: Cervical adenosquamous carcinoma.

## Results

### Clinicopathological characteristics of the study cohort

As shown in Figure 1, 497 patients with ASC who underwent radical hysterectomy were enrolled in this study. All eligible participants were randomly assigned to either the training cohort (348 patients) or the validation cohort (149 patients) at a ratio of approximately 7:3. Detailed clinicopathologic features and disease characteristics of the patients are shown in Table 1. There were 57.5% (*n* ═ 200) and 58.4% (*n* ═ 87) of patients aged ≥ 46 years in the training and validation cohorts, respectively. The predominant ethnic group in both cohorts was white, constituting 78.2% in the training group and 68.5% in the validation group. The distribution of histologic grades I and III was 48.6% and 22.7% in the training cohort and 49.0% and 23.5% in the validation cohort. Tumors exceeding 4 cm in diameter were observed in approximately 40.8% and 40.9% of patients in the training and validation cohorts, respectively. The T-, N-, and M-stages predominantly fell into the T1 + T2 stage (83.0%, 79.2%), N0 stage (87.6%, 83.2%), and M0 stage (94.8%, 91.9%) in both the training and validation cohorts. Regarding treatment modalities, most patients in both groups underwent radiotherapy, accounting for 65.8% in the training cohort and 61.1% in the validation cohort. Chemotherapy was administered to 53.4% of patients in the training cohort and 53.0% in the validation cohort. The chi-square or Fisher’s exact test revealed no discernible differences between the training and validation groups (all *P* > 0.05).

### Probability of cancer-specific death in ASC patients treated with radical hysterectomy

The median follow-up time for the entire study was 73 months (range: 1–145 months). During the follow-up, 140 patients died, with 106 (75.7%) experiencing mortality attributable to ASC and the remaining 34 (24.3%) succumbing to other causes.

**Figure 2.**
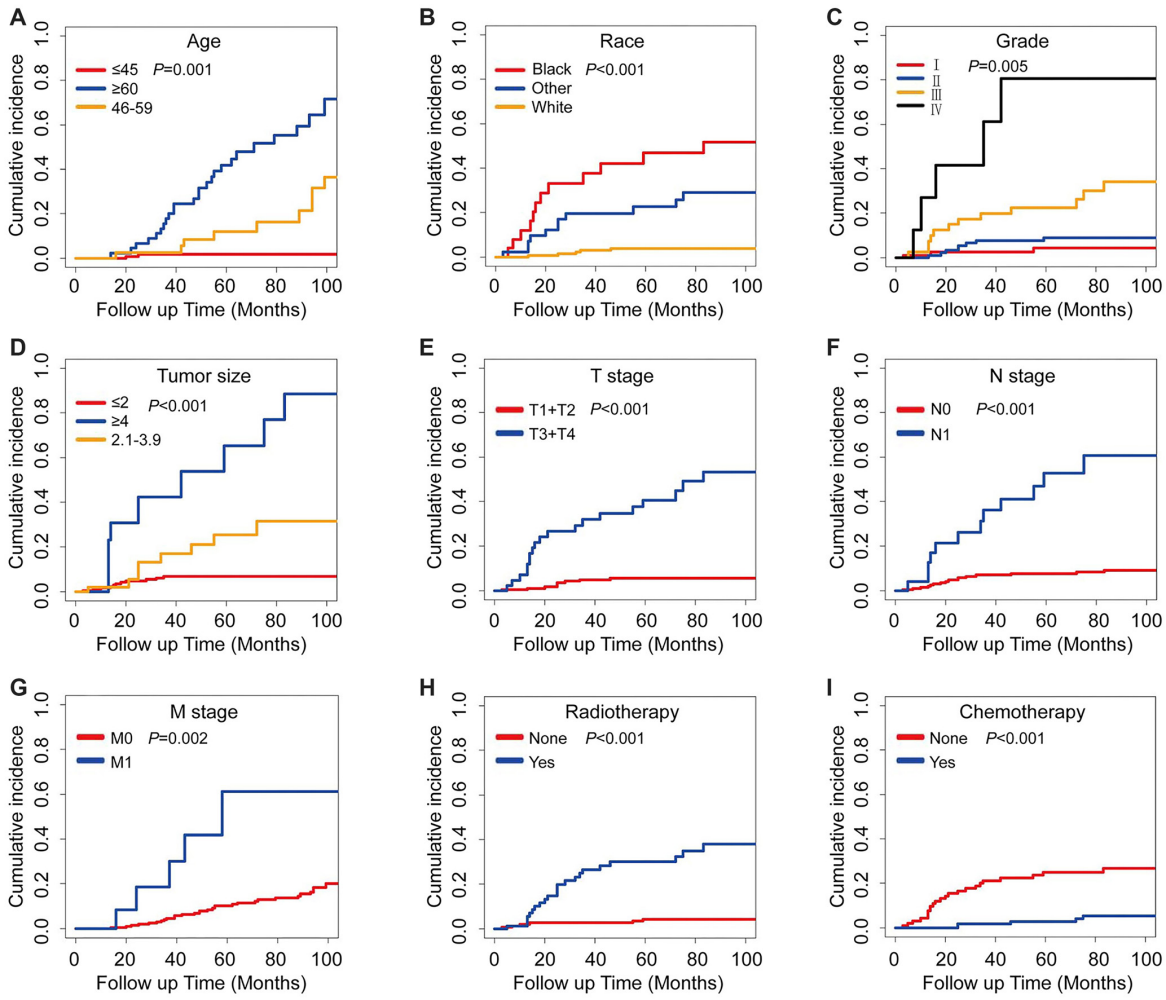
**Cumulative incidence functions of ASC-specific death based on different key characteristics.** The *P* values for each variable were determined using Gray’s test. ASC: Cervical adenosquamous carcinoma.

Table 2 shows the cumulative cancer-specific mortality rates at 1-, 3-, and 5-year intervals for ASC patients who underwent radical hysterectomy, categorized by distinct clinicopathological features. Corresponding CIF curves are presented in Figure 2. Overall, the 1-, 3-, and 5-year cancer-specific mortality rates for ASC patients undergoing radical hysterectomy were 5.4%, 13.1%, and 28.7%, respectively. Furthermore, the outcomes highlighted positive associations between higher cancer-specific mortality and certain characteristics, such as older age, black race, higher grade, larger tumors, advanced TNM stage, and the receipt of radiotherapy without chemotherapy. Notably, patients with grade IV exhibited the highest probability of succumbing to a specific cause (1-year: 27.1%; 3-year: 61.1%; and 5-year: 80.6%).

**Table 2:** Cumulative incidence of ASC-specific death at 1-, 3-, and 5-year intervals in ASC patients treated with radical hysterectomy

**Variables**	**Patients**		**Cancer-specific mortality(%)**			***P^‡^* value**
	**No.**	**%**	**1-year**	**3-year**	**5-year**	
Total	348	100	5.4	13.1	28.7	
*Age (year)*						**0.001**
≤ 45	148	42.5	0.3	1.6	1.6	
46–59	110	31.6	0.1	2.5	12.1	
≥ 60	90	25.9	0.5	18.3	41.9	
*Race*						**<0.001**
Black	28	8	12	37.7	47.1	
White	272	78.2	0.3	2.9	3.7	
Other	48	13.8	2.3	19.7	22.6	
*Grade*						**0.005**
I	169	48.6	1.1	2.4	4.5	
II	56	16.1	0.2	7.6	8.8	
III	79	22.7	2.4	19.9	22.5	
IV	44	12.6	27.1	61.1	80.6	
*Tumor size (cm)*						**<0.001**
≤ 2	90	25.9	1.8	6.7	6.7	
2.1–3.9	116	33.3	2.1	17	25.1	
≥ 4	142	40.8	0.6	42.3	65.4	
*T stage*						**<0.001**
T1 + T2	289	83	0.5	4.9	5.5	
T3 + T4	59	17	7	32	40.6	
*N stage*						**<0.001**
N0	305	87.6	1.5	7	7.6	
N1	43	12.4	4	36.2	52.9	
*M stage*						**0.002**
M0	330	94.8	0.2	4.8	10.3	
M1	18	5.2	0.4	18.5	61.3	
*Radiotherapy*						**<0.001**
None	119	34.2	2	2.7	4.3	
Yes	229	65.8	1.3	26.6	30.2	
*Chemotherapy*						**<0.001**
None	162	46.6	4.2	21.2	25.1	
Yes	186	53.4	0.1	1.8	2.7	
*Marital status*						0.735
Single	190	54.6	1.5	10.9	12.8	
Married	158	45.4	2	9	11.6	

*P*^‡^ indicates Gray’s test. ASC: Cervical adenosquamous carcinoma.

### Construction of competing risk nomogram for ASC patients treated with radical hysterectomy

**Figure 3.**
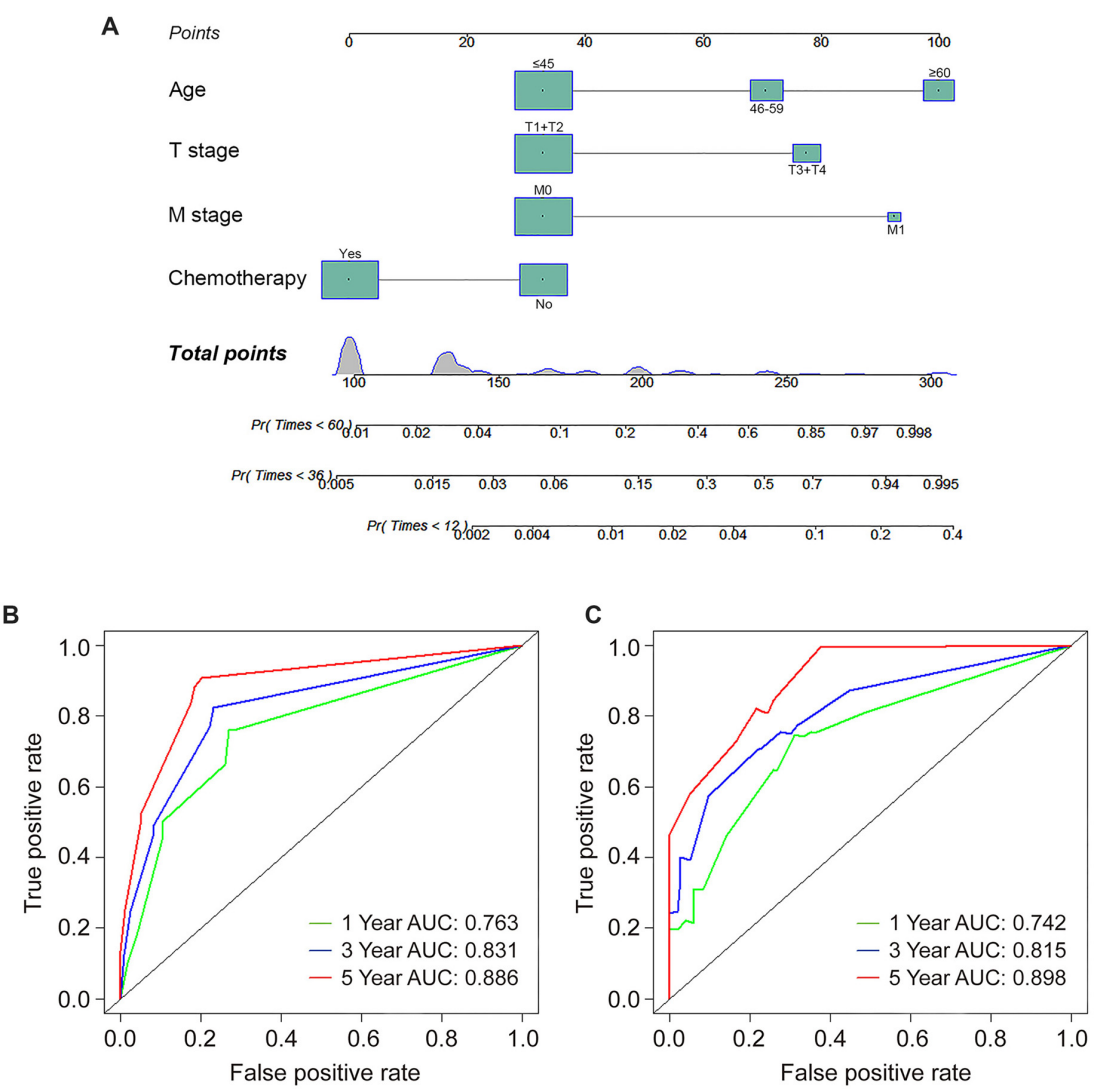
Competing risk nomogram predicting the 1-, 3-, and 5-year cumulative incidence of ASC-specific death for ASC patients treated with radical hysterectomy (A). ROC curves depicting the 1-, 3-, and 5-year predicted cumulative incidence of ASC-specific death for ASC patients undergoing radical hysterectomy in (B) the training cohort and (C) the validation cohort. ASC: Cervical adenosquamous carcinoma; ROC: Receiver operating characteristic.

To identify independent predictors associated with ASC-specific mortality, all candidate variables were included in a univariate competing risk analysis. Significant variables from the univariate analysis were further examined using the Fine–Gray model. Independent predictors of ASC-specific mortality included age (relative to ≤ 45: subdistribution hazard ratio [SHR]: 3.524, 95% CI: 2.046–5.002, *P* ═ 0.037 for 46–59; SHR: 9.631, 95% CI: 7.973–11.289, *P* ═ 0.012 for ≥ 60), T stage (SHR: 4.246, 95% CI: 3.105–5.387, *P* ═ 0.016), M stage (SHR: 7.746, 95% CI: 6.512–8.980, *P* ═ 0.005), and chemotherapy (SHR: 0.134, 95% CI: 0.052–0.216, *P* ═ 0.022) (Table 3). A multivariate Cox proportional hazards model was also applied for comparative analysis. The Cox regression analysis identified age (relative to ≤ 45: hazard ratio [HR]: 4.023, 95% CI: 3.506–4.540, *P* ═ 0.034 for 46–59; HR: 10.147, 95% CI: 8.658–11.636, *P* ═ 0.003 for ≥ 60), grade (relative to stage I: HR: 4.527, 95% CI: 3.603–5.451, *P* ═ 0.048 for stage III; HR: 10.593, 95% CI: 9.814–11.372, *P* ═ 0.032 for stage IV), T stage (HR: 5.436, 95% CI: 4.312–6.560, *P* ═ 0.002), M stage (HR: 9.489, 95% CI: 8.323–10.655, *P* < 0.001), and chemotherapy (HR: 0.125, 95% CI: 0.044–0.206, *P* ═ 0.013) as independent predictors (Table 3). Subsequently, a competing risk nomogram was constructed, incorporating the four identified independent predictors (Figure 3A). Within the competing risk nomogram, age and M stage had the most substantial influence on ASC-specific mortality, followed by T stage and chemotherapy. The nomogram facilitated the calculation of total scores by summing the specific points associated with each predictor. The 1-, 3-, and 5-year predicted cumulative incidence of ASC-specific death corresponding to the total points scale were delineated at the base of the competing risk nomogram.

**Table 3:** Multivariate analyses using the subdistribution proportional hazards model and Cox regression model

**Variables**	**Subdistribution proportional hazards model**			**Cox regression model**		
	**Coefficient**	**SHR (95% CI)**	***P^§^* value**	**Coefficient**	**HR (95% CI)**	***P^ɛ^* value**
*Age (year)*						
≤ 45		Reference			Reference	
46–59	1.142	3.524 (2.046, 5.002)	**0.037**	1.186	4.023 (3.506, 4.540)	**0.034**
≥ 60	1.998	9.631 (7.973, 11.289)	**0.012**	2.066	10.147(8.658,11.636)	**0.003**
*Race*						
Black		Reference			Reference	
White	−0.411	0.536 (0.023, 1.049)	0.252	−0.460	0.519 (0.021, 1.017)	0.236
Other	−0.333	0.620 (0.187, 1.053)	0.304	−0.358	0.587 (0.108, 1.066)	0.285
*Grade*						
I		Reference			Reference	
II	1.016	1.735 (0.264, 3.206)	0.074	1.038	2.062 (0.598, 3.526)	0.065
III	1.094	3.217 (0.901, 5.533)	0.061	1.263	4.527 (3.603, 5.451)	**0.048**
IV	1.897	8.066 (0.984, 15.148)	0.051	2.121	10.593 (9.814, 11.372)	**0.032**
*Tumor size (cm)*						
≤ 2		Reference			Reference	
2.1–3.9	1.058	2.063 (0.340, 3.786)	0.068	1.171	3.574 (0.861, 6.287)	0.063
≥ 4	1.309	5.791 (0.825, 10.757)	0.055	1.415	6.145 (0.983, 11.307)	0.052
*T stage*						
T1 + T2		Reference			Reference	
T3 + T4	1.285	4.246 (3.105,5.387)	**0.016**	1.334	5.436 (4.312,6.560)	**0.002**
*N stage*						
N0		Reference			Reference	
N1	1.360	6.427 (0.893, 11.961)	0.074	1.464	7.126 (0.905, 13.347)	0.067
*M stage*						
M0		Reference			Reference	
M1	1.753	7.746 (6.512, 8.980)	**0.005**	1.847	9.489 (8.323,10.655)	**<0.001**
*Radiotherapy*						
None		Reference			Reference	
Yes	1.043	2.046 (0.094, 3.998)	0.151	1.122	3.117 (0.160, 6.074)	0.136
*Chemotherapy*						
None		Reference			Reference	
Yes	-0.517	0.134 (0.052, 0.216)	**0.022**	-0.623	0.125(0.044, 0.206)	**0.013**
*Marital status*						
Single		Reference			Reference	
Married	-0.293	0.663 (0.210, 1.116)	0.587	-0.406	0.579 (0.113, 1.045)	0.484

*P*^§^ indicates Fine–Gray test, *P*^ɛ^ indicates Wald test. SHR: Subdistribution hazard ratio; HR: Hazard ratio.

### Validation and clinical value of competing risk nomogram

**Figure 4.**
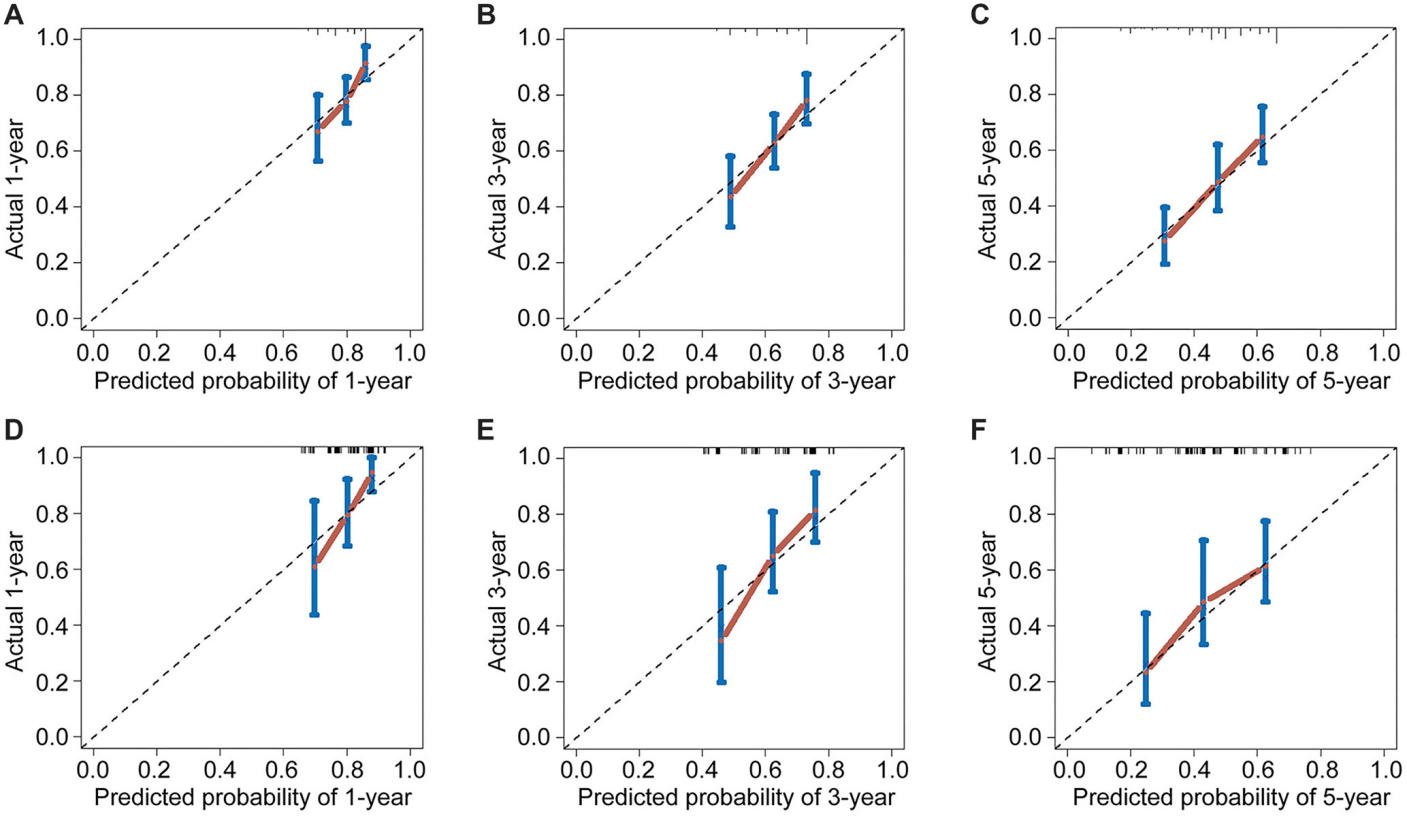
Calibration curves for the nomogram illustrating the (A) 1-, (B) 3-, and (C) 5-year predicted cumulative incidence of ASC-specific death for ASC patients treated with radical hysterectomy in the training cohort. Additionally, calibration curves for predicting the (D) 1-, (E) 3-, and (F) 5-year cumulative incidence of ASC-specific death for ASC patients treated with radical hysterectomy in the validation cohort. ASC: Cervical adenosquamous carcinoma.

**Figure 5.**
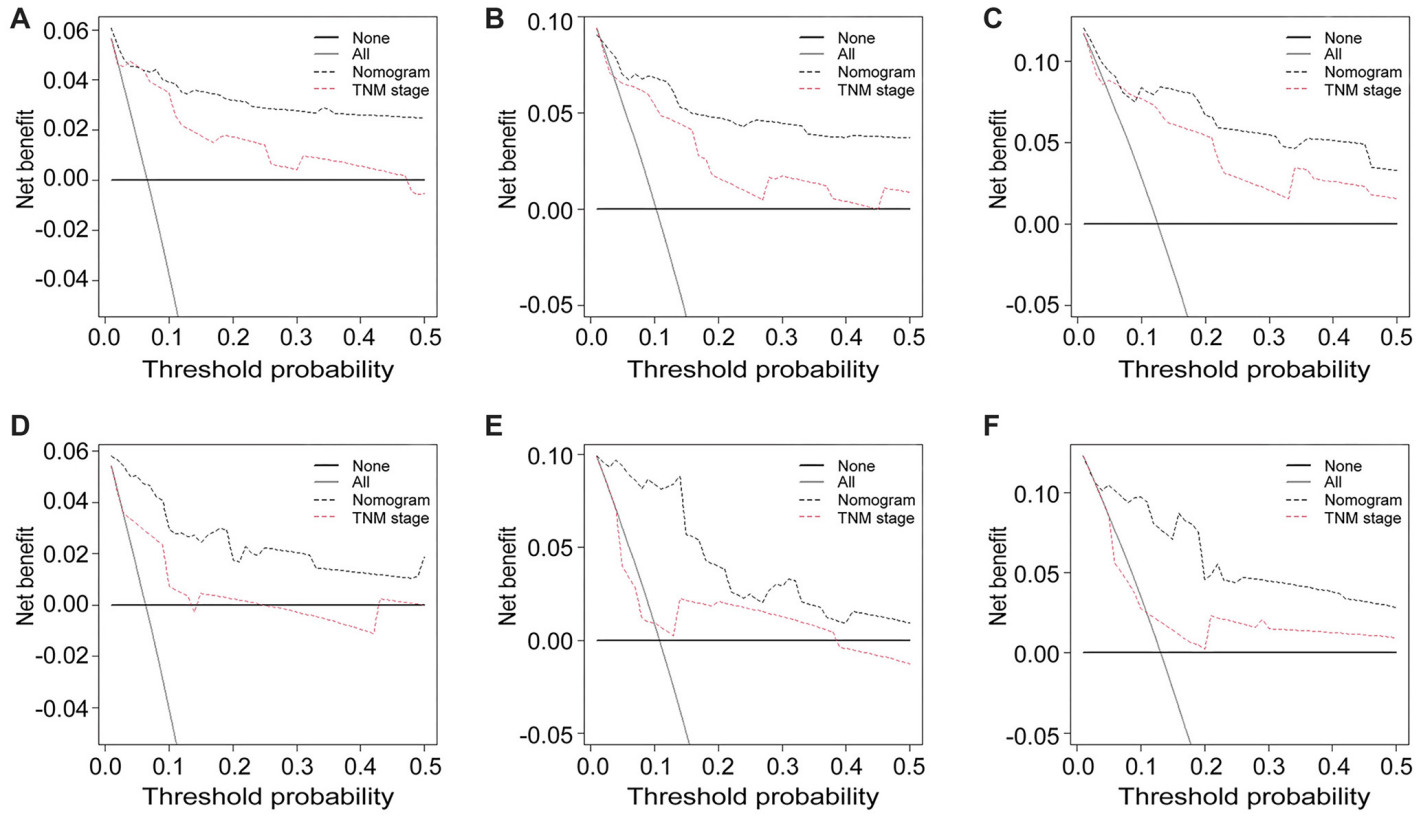
DCA illustrates the clinical benefits of the nomogram in predicting the (A) 1-, (B) 3-, and (C) 5-year cumulative incidence of ASC-specific death for ASC patients subjected to radical hysterectomy in the training cohort. Furthermore, DCA results for predicting the (D) 1-, (E) 3-, and (F) 5-year cumulative incidence of ASC-specific death for ASC patients treated with radical hysterectomy in the validation cohort. DCA: Decision curve analysis; ASC: Cervical adenosquamous carcinoma.

**Figure 6.**
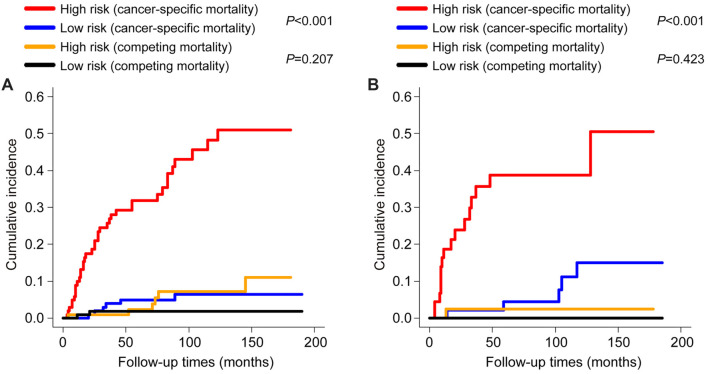
Cumulative incidence function curves with associated *P* values from Gray’s test comparing the high-risk and low-risk groups in (A) the training cohort and (B) the validation cohort. The *P* values were determined using the log-rank test.

The competing risk nomogram demonstrated a C-index of 0.873 (95% CI: 0.852–0.894) for the training cohort and 0.825 (95% CI: 0.809–0.841) for the validation cohort. This contrasts with the C-indices of the TNM staging system, which were 0.651 (95% CI: 0.628–0.674) in the training cohort and 0.607 (95% CI: 0.590–0.624) in the validation cohort. Additionally, ROC analysis revealed that the nomogram’s area under the ROC curves (AUCs) for predicting the 1-, 3-, and 5-year cumulative incidence of ASC-specific death were 0.763 (95% CI: 0.667–0.859), 0.831 (95% CI: 0.808–0.854), and 0.886 (95% CI: 0.801–0.971) in the training cohort, and 0.742 (95% CI: 0.633–0.851), 0.815 (95% CI: 0.783–0.847), and 0.898 (95% CI: 0.804–0.992) in the validation cohort, respectively (Figure 3B and 3C). The 3-year AUC did not differ significantly from the 1-year AUC in either the training or validation sets (*P* ═ 0.083 and *P* ═ 0.107, respectively), whereas the 5-year AUC differed from the 1-year AUC in both the training and validation sets (*P* ═ 0.041 and *P* ═ 0.032, respectively). These findings underscored the excellent discriminatory ability of the competing risk nomogram, effectively distinguishing ASC-specific mortality from other-cause mortality and censored events. The calibration curves for the competing risk nomogram, depicting the predicted cumulative incidence of ASC-specific death in ASC patients treated with radical hysterectomy at 1-, 3-, and 5-year intervals (Figure 4A–4C), demonstrated a close fit to the 45∘ ideal line in the training cohort. This alignment suggested consistent concordance between predicted mortality from ASC and observed mortality. Similarly, the calibration curves for the nomogram predicting the 1-, 3-, and 5-year cumulative incidence of ASC-specific death (Figure 4D–4F) exhibited robust agreement between predictions and actual observations in the validation cohort. Decision curves, representing the clinical benefits of the competing risk nomogram, were further plotted. The DCA curves illustrated strong positive net clinical benefits across various threshold probabilities for predicting the 1-, 3-, and 5-year cumulative incidence of ASC-specific death when compared to the traditional TNM staging system in both the training and validation cohorts (Figure 5). Moreover, a corresponding risk classification system was constructed. Participants were categorized into high-risk (total score ≥ 206) and low-risk (total score < 206) groups based on the dichotomy values of the nomogram-based scores. In both the training and validation sets, the high-risk group exhibited higher probabilities of ASC-specific death than the low-risk group (Figure 6).

## Discussion

ASC represents a rare subtype of cervical cancer, and current prognostic studies on ASC often rely on limited case analysis. The competing risk model, distinguished by its incorporation of competing events into the analysis, surpasses traditional methodologies, such as the Kaplan–Meier analysis and Cox regression models. This model facilitates a nuanced differentiation between the impacts of treatment and risk factors on specific events [17–19]. In this study, we established an innovative competing risk model, leveraging a sizable dataset from the SEER database. This model predicts factors influencing cancer-specific mortality in patients with ASC who underwent radical hysterectomy. Throughout the follow-up period, 140 out of 497 ASC patients subjected to radical hysterectomy died, with 106 (75.7%) deaths attributed to ASC and 34 (24.3%) to other causes. The 1-, 3-, and 5-year cancer-specific mortality rates for ASC patients following radical hysterectomy were 5.4%, 13.1%, and 28.7%, respectively. Older patients, those who were black, with higher grade, larger tumors, advanced TNM stage, and those who received radiotherapy but not chemotherapy demonstrated a higher cumulative incidence of cause-specific death. The multivariate competing risk model indicated that age, T stage, M stage, and chemotherapy are significant independent predictors for ASC-specific mortality. Unlike the competing risk model, the Cox regression model identified grade as a significant prognostic factor. However, comparing the two models highlights a key issue: the Cox regression model overestimates the HR for most variables and may incorrectly identify grade as a significant prognostic factor due to false positives. The competing risk model facilitates the elimination of confounding factors associated with mortality from other causes, a crucial step for precision in deploying optimal clinical intervention strategies. The development of the competing risk nomogram involved the integration of these four independent predictors, aiming to forecast the 1-, 3-, and 5-year cumulative incidences of ASC-specific death. Notably, age and M stage exhibited the most substantial contributions to ASC-specific mortality, followed by T stage and chemotherapy.

Our study revealed an escalating probability of ASC-specific death with advancing age. This observation may be attributed, on the one hand, to the heightened prevalence of comorbidities, such as hypertension and diabetes among older individuals, posing significant threats to the survival of ASC patients in this age group. On the other hand, the absence of hormonal protection in postmenopausal women may further contribute to the increased incidence of ASC-specific death. Lichter et al. [20] highlighted that cervical cancer patients over 80 years old predominantly presented with stage IV disease, a complication score of ≥ 3, and seldom received effective treatment, leading to a reduction in five-year cancer-specific survival to 50%. Moreover, in a separate retrospective study, Jiang and Cai [21] indicated that older patients with neuroendocrine cervical carcinoma faced an elevated risk of cancer-specific mortality (HR ═ 1.659, 95% CI: 1.091–2.524, *P* ═ 0.018).

This study observed a notable increase in ASC-specific mortality rates in patients with higher T and M stages. The T stage effectively reflects intrinsic tumor properties, wherein an elevated T stage correlates with a greater tumor burden, consequently contributing to heightened tumor-specific mortality. The M1 stage suggests distant metastasis, placing patients at an advanced disease stage and, consequently, a poorer prognosis. Ni et al. [22] underscored the pivotal role of advanced T stage in the specific mortality of uterine cervical adenocarcinoma (HR ═ 14.10, 95% CI: 8.77–22.69, *P* < 0.001), with a significant increase in mortality observed for patients with M1 stage as well (HR ═ 1.71, 95% CI: 1.44–2.03, *P* < 0.001). Similarly, Xu et al. [23] highlighted higher mortality rates and a worse prognosis for patients with high T and M stages in gastric signet ring cell carcinoma (T4 vs T1 HR ═ 5.364, 95% CI: 2.861–10.057, *P* < 0.001; M1 vs M0 HR ═ 2.050, 95% CI: 1.514–2.775, *P* < 0.001).

Regarding post-radical hysterectomy treatment options for ASC, our observations revealed that systemic adjuvant chemotherapy, as opposed to local radiotherapy, significantly reduces the incidence of ASC-specific death. When comparing radiotherapy and non-radiotherapy groups, no significant differences were observed between the T, N, and M stages (all *P* > 0.05). However, the cumulative incidence of ASC-specific mortality was higher in the radiotherapy group compared to the non-radiotherapy group, a finding that may be attributed to a complex interplay of factors. Firstly, radiotherapy induces substantial non-specific damage to surrounding normal tissue cells, which can impair physiological functions and potentially accelerate disease progression. Secondly, the combination of radiotherapy and surgery may increase the risk of complications due to their synergistic effects, thereby negatively impacting patient prognosis [24]. Additionally, radiation-induced double-strand DNA breaks and subsequent cellular DNA damage lead to direct cell killing and can cause gene mutations, potentially resulting in the malignant transformation of irradiated cells and the induction of secondary tumors [25]. These challenges to long-term survival may contribute to an elevated mortality rate. Tong et al. [26] found that patients with secondary uterine malignancies who received radiotherapy had a 1.6-fold higher risk of death compared to those who did not (HR: 2.6, 95% CI: 1.407–4.804, *P* ═ 0.002), with a shorter median survival time and a poorer prognosis. Wu et al. [27] reported that cervical cancer patients treated with radiotherapy had a significantly increased risk of developing second primary cancers in the colon, rectum, and anus (HR: 1.43; 95% CI: 1.09–1.87; *P* ═ 0.01), lungs and bronchi (HR: 1.41; 95% CI: 1.13–1.76; *P* ═ 0.002), uterus (HR: 3.71; 95% CI: 1.71–8.06; *P* < 0.001), ovaries (HR: 2.79; 95% CI: 1.38–5.64; *P* ═ 0.004), and bladder (HR: 2.18; 95% CI: 1.35–3.54; *P* ═ 0.002). Adjuvant chemotherapy following radical hysterectomy is considered an effective regimen for locally advanced cervical cancer and is widely incorporated into clinical practice. Feng et al. [28] reported that surgery followed by three cycles of adjuvant chemotherapy significantly enhances clinical outcomes for the best responders in terms of DFS, OS, and drug toxicity in locally advanced cervical cancer patients. Furthermore, Georgescu et al. reported a case of a 32-year-old female with metastatic ASC, whose prognosis was unfavorable. However, a multimodal treatment approach was applied to this case, involving concurrent CT-based image-guided brachytherapy with paclitaxel and carboplatin chemotherapy, followed by radical hysterectomy once the tumor achieved complete or partial response, or disease stability. Subsequently, adjuvant chemotherapy was incorporated into the treatment plan postoperatively, which led to effective local control and prolonged survival [29].

The competing risk nomogram, encompassing four easily accessible variables—age, T stage, M stage, and chemotherapy—has been developed to aid clinicians in making informed assessments within routine clinical practice. The established nomogram demonstrated optimal calibration and precise discrimination capabilities, yielding C-indices of 0.873 (95% CI, 0.852–0.894) and 0.825 (95% CI, 0.809–0.841) for the training and validation cohorts, respectively. In comparison, the TNM staging system yielded significantly lower C-indices of 0.651 (95% CI, 0.628–0.674) in the training cohort and 0.607 (95% CI, 0.590–0.624) in the validation cohort. Additionally, the DCA confirmed the enhanced clinical benefit of the nomogram over the traditional TNM staging system. These findings suggest that the competing risk model has superior predictive performance compared to the TNM staging system. For instance, a 49-year-old woman diagnosed with ASC and without distant metastases on CT scans underwent radical hysterectomy. Biopsy results revealed a T2N1M0 stage and poorly differentiated cell type. According to the nomogram, age 49 corresponds to 71 points, T2 to 32 points, and M0 to 32 points. If chemotherapy is administered post-surgery, the score is 0 points, totaling 135 points, with 1-year, 3-year, and 5-year ASC-specific mortality rates of less than 0.2%, 2%, and 3.3%, respectively. Conversely, if chemotherapy is not administered post-surgery, the score is 32 points, totaling 167 points, with 1-year, 3-year, and 5-year ASC-specific mortality rates of 0.54%, 5.8%, and 9.6%, respectively. Therefore, based on the predicted results, the patient is recommended to undergo three cycles of cisplatin chemotherapy post-radical hysterectomy to prolong survival. Additionally, patients were stratified into two groups based on dichotomy values derived from the nomogram-based scores, revealing higher probabilities of ASC-specific death in the high-risk group compared to the low-risk group in both the training and validation sets. The competing risk nomogram enables clinicians to effectively distinguish between various risk groups, facilitating comprehensive treatment strategies and vigilant monitoring for high-risk patients.

### Limitations

While this study represents the first effort to develop and validate a competing risk nomogram for predicting cancer-specific death in ASC patients undergoing radical hysterectomy, based on the extensive SEER database, several limitations warrant consideration. First, being retrospective in nature, the exclusion of patients with incomplete data for the inclusion criteria introduced inevitable selection bias. Second, certain pivotal prognostic variables, including chemotherapy regimens, radiotherapy dosage, and lymphovascular invasion, were unavailable in the SEER database, thereby constraining further analysis. The patients in our study were diagnosed between 2010 and 2019, resulting in a relatively short follow-up period. Including these variables and extending the follow-up period could potentially enhance the predictive efficacy of the nomogram. Third, the data used to establish and validate the competing risk nomogram were sourced from the same research center, and our study specifically focused on ASC patients undergoing radical hysterectomy. The absence of comparisons with alternative treatments restricts the generalizability of our nomogram. Acknowledging these limitations, comprehensive validation through multicenter prospective clinical trials is needed to substantiate the accuracy of this predictive tool.

## Conclusion

This study, leveraging a substantial population from the SEER database, represents the first endeavor to establish a competing risk model for predicting ASC-specific mortality in ASC patients undergoing radical hysterectomy. The model proves to be an accurate and clinically valuable tool, enabling clinicians to optimize treatment plans and formulate desirable, personalized follow-up strategies for ASC patients in an effective and convenient manner.

## Data Availability

Publicly available SEER datasets were used in the present study. The data can be found at https://seer.cancer.gov/data/.
